# Missing value imputation in high-dimensional phenomic data: imputable or not, and how?

**DOI:** 10.1186/s12859-014-0346-6

**Published:** 2014-11-05

**Authors:** Serena G Liao, Yan Lin, Dongwan D Kang, Divay Chandra, Jessica Bon, Naftali Kaminski, Frank C Sciurba, George C Tseng

**Affiliations:** Department of Biostatistics, University of Pittsburgh, Pittsburgh, PA USA; Department of Computational and Systems Biology, University of Pittsburgh, Pittsburgh, PA USA; Department of Human Genetics, University of Pittsburgh, Pittsburgh, PA USA; Pulmonary, Critical Care and Sleep Medicine, Yale School of Medicine, New Haven, CT USA; Department of Medicine, University of Pittsburgh, Pittsburgh, PA USA

**Keywords:** Missing data, K-nearest-neighbor, Phenomic data, Self-training selection

## Abstract

**Background:**

In modern biomedical research of complex diseases, a large number of demographic and clinical variables, herein called phenomic data, are often collected and missing values (MVs) are inevitable in the data collection process. Since many downstream statistical and bioinformatics methods require complete data matrix, imputation is a common and practical solution. In high-throughput experiments such as microarray experiments, continuous intensities are measured and many mature missing value imputation methods have been developed and widely applied. Numerous methods for missing data imputation of microarray data have been developed. Large phenomic data, however, contain continuous, nominal, binary and ordinal data types, which void application of most methods. Though several methods have been developed in the past few years, not a single complete guideline is proposed with respect to phenomic missing data imputation.

**Results:**

In this paper, we investigated existing imputation methods for phenomic data, proposed a self-training selection (STS) scheme to select the best imputation method and provide a practical guideline for general applications. We introduced a novel concept of “imputability measure” (IM) to identify missing values that are fundamentally inadequate to impute. In addition, we also developed four variations of K-nearest-neighbor (KNN) methods and compared with two existing methods, multivariate imputation by chained equations (MICE) and missForest. The four variations are imputation by variables (KNN-V), by subjects (KNN-S), their weighted hybrid (KNN-H) and an adaptively weighted hybrid (KNN-A). We performed simulations and applied different imputation methods and the STS scheme to three lung disease phenomic datasets to evaluate the methods. An R package “phenomeImpute” is made publicly available.

**Conclusions:**

Simulations and applications to real datasets showed that MICE often did not perform well; KNN-A, KNN-H and random forest were among the top performers although no method universally performed the best. Imputation of missing values with low imputability measures increased imputation errors greatly and could potentially deteriorate downstream analyses. The STS scheme was accurate in selecting the optimal method by evaluating methods in a second layer of missingness simulation. All source files for the simulation and the real data analyses are available on the author’s publication website.

**Electronic supplementary material:**

The online version of this article (doi:10.1186/s12859-014-0346-6) contains supplementary material, which is available to authorized users.

## Background

In many studies of complex diseases, a large number of demographic, environmental and clinical variables are collected and missing values (MVs) are inevitable in the data collection process. Major categories of variables include but not limited to: (1) demographic measures, such as gender, race, education and marital status; (2) environmental exposures, such as pollen, feather pillows and pollutions; (3) living habits, such as exercise, sleep, diet, vitamin supplement and smoking; (4) measures of general health status or organ function, such as body mass index (BMI), blood pressure, walking speed and forced vital capacity (FVC); (5) summary measures from medical images, such as fMRI and PET scan; (6) drug history; and (7) family disease history. The dimension of the data can easily go beyond several hundreds to nearly a thousand and we refer to such data as “phenomic data”, hereafter. It has been shown recently that systematic analysis of the phenomic data and integration with other genomic information provide further understanding of diseases [1-5], and enhance disease subtype discovery towards precision medicine [6,7]. The presence of missing values in clinical research not only reduces statistical power of the study but also impedes the implementation of many statistical and bioinformatic methods that require a complete dataset (e.g. principal component analysis, clustering analysis, machine learning and graphical models). Many have pointed out that “missing value has the potential to undermine the validity of epidemiologic and clinical research and lead the conclusion to bias” [8].

Standard statistical methods for analysis of data with missing values include list-wise deletion or complete-case analysis (i.e. discard any subject with a missing value), likelihood-based methods, data augmentation and imputation [9,10]. The list-wise deletion in general leads to loss of statistical power and biased results when data are not missing completely at random. Likelihood-based methods and data augmentation are popular for low dimensional data with parametric models for the missing-data process [10,11]. However, their application in high dimensional data is problematic especially when the missing data pattern is complicated and the required intensive computing is most likely insurmountable. On the contrary, imputation provides an intuitive and powerful tool for analysis of data with complex missing-data patterns [12-16]. Explicit imputation methods such as mean imputation or stochastic imputation either undermines the variability of the data or requires parametric assumption on the data and subsequently faces similar challenges as the likelihood-based method and data augmentation [12-14,16]. Implicit imputation methods such as nearest-neighbour imputation, hot-deck and fractional imputation provide flexible and powerful approaches for analysis of data with complex missing-data patterns even though the implicit imputation model is not coherent with the assumed model for the underlying complete data [13,17,18]. Multiple imputations usually are considered to account for the variability due to imputation [13,14,16,19].

Except for some implicit imputation methods, other above-mentioned methods rely on correct modelling of the missing data process and work well in traditional situations with large number of subjects and small number of variables (large n, small p). With the trend of increasing number of variables (large p) in phenomic data, the model fitting, diagnostic check and sensitivity analysis become difficult to ensure success of multiple imputation or maximum likelihood imputation. The complexity of phenomic data with mixed data types (binary, multi-class categorical, ordinal and continuous) further aggravates the difficulties of modeling the joint distribution of all variables. Although a few of the algorithms are designed to handle datasets with both continuous and categorical variables [14,20-22], the implementation of most of these complicated methods in the high dimensional phenomic data is not straightforward. Imputation methods by exact statistical modeling often suffer from “curse of dimensionality”. Jerez and colleagues compared machine learning methods, such as multi-layer perceptron (MLP), self-organizing maps (SOM) and k-nearest neighbor (KNN), to traditional statistical imputation methods in a large breast cancer dataset and concluded that machine learning imputation methods seemed to perform better in this large clinical data [23].

In the past decade, missing value imputation for high-throughput experimental data,(e.g. microarray data) has drawn great attention and many methods have been developed and widely used (see [24], [25] for review and comparative studies). Imputation of phenomic data differs from microarray data and brings new challenges for two major reasons. Firstly microarray data contain entirely continuous intensity measurements, while phenomic data have mixed data types. This voids majority of established microarray imputation methods for phenomic data. Secondly, microarray data monitor gene expression of thousands of genes and the majority of the genes are believed to be co-regulated with others in a systemic sense, which leads to a highly correlated structure of the data and makes imputation intrinsically easier. The phenomic data, on the other hand, are more likely to contain isolated variables (or samples) that are “not imputable” from other observed variables (samples).

There are at least three aspects of novelty in this paper. Firstly, to our knowledge, this is the first systematic comparative study of missing value imputation methods for large-scale phenomic data. We will compare two existing methods (missForest [26] and multivariate imputation by chained equations, MICE [16]) and extend four variants of KNN imputation method that was popularly used in microarray analysis [27]. Secondly, to characterize and identify missing values that are “not imputable” from other observed values in phenomic data, we propose an “imputability measure” (IM) to quantify imputability of a missing value. When a variable or subject has an overall small IM in its missing values, it is recommended to remove the variable or subject from further analysis (or impute with caution). Thirdly, we propose a self-training scheme (STS) [24] to select the best missing value imputation method for each data type in a given dataset. The result provides a practical guideline in applications. The IM and STS selection tool will remain useful when more powerful methods for phenomic data imputation are developed in the future.

## Methods

### Real data

The current work is motivated by three high-dimensional phenomic datasets, all of which have a mixture of continuous, ordinal, binary and nominal covariates. The Chronic Obstructive Pulmonary Disease (COPD) dataset was generated from a COPD study conducted in the Division of Pulmonary, Department of Medicine at the University of Pittsburgh. The second dataset is the phenotypic data set of the Lung Tissue Research Consortium (LTRC, http://www.nhlbi.nih.gov/resources/ltrc.htm). The third dataset is obtained from the Severe Asthma Research Program (SARP) study (http://www.severeasthma.org/). These datasets represent different variable/subject ratios and different proportions of data types in the variables. In Table 1, Raw Data (RD) refers to the original raw data with missing values we initially obtained. Complete Data (CD) represents a complete dataset without any missing value after we iteratively remove variables and subjects with large missing value percentage. CDs contain no missing values and are ideal to perform simulation for evaluating different methods (see section Simulated datasets).

**Table 1:** **Descriptions of three real data sets**

**Number of variables and subjects**	**COPD**	**LTRC**	**SARP**
Subjects (RD/CD)	699/491	1428/709	1671/640
Variables (RD/CD)	528/257	1568/129	1761/135
Continuous variables (Con)	113	11	27
Multi-class categorical variables (Cat)	12	27	6
Binary variables (Bin)	78	0	86
Ordinal variables (Ord)	54	91	16
Total variables in CD	257	129	135

### Imputation methods

We will compare four newly developed KNN methods with the MICE and the missForest methods in this paper. The methods and detailed implementations are described below.

#### Two existing methods MICE and missForest

Multivariate Imputation by Chained Equations (MICE) is a popular method to impute multivariate missing data. It factorizes the joint conditional density as a sequence of conditional probabilities and imputes missing values by multiple regression sequentially based on different types of missing covariates. Gibbs sampling is used to estimate the parameters. It then draws imputation for each variable condition on all the other variables. We used the R package “MICE” to implement this method.

MissForest is a random forest based method to impute phenomic data [26]. The method treats the variable of the missing value as the response variable and borrows information from other variables by the resampling-based classification and regression trees to grow a random forest for the final prediction. The method is repeated until the imputed values reach convergence. The method is implement in the “missForest” R package.

#### KNN imputation methods

KNN method is popular due to its simplicity and proven effectiveness in many missing value imputation problems. For a missing value, the method seeks its K nearest variables or subjects and imputes by a weighted average of observed values of the identified neighbours. We adopted the weight choice from the LSimpute method used for microarray missing value imputation [28]. LSimpute is an extension of the KNN, which utilizes correlations between both genes and arrays, and the missing values are imputed by a weighted average of the gene and array based estimates. Specifically, the weight for the k^th^ neighbor of a missing variable or subject was given by ${\mathrm{w}}_{\mathrm{k}}={\left({\mathrm{r}}_{\mathrm{k}}^2/\left(1-{\mathrm{r}}_{\mathrm{k}}^2+\upvarepsilon \right)\right)}^2$, where r_k_ is the correlation between the k^th^ neighbor and the missing variable or subject and ε = 10^− 6^. As a result, this algorithm gives more weight to closer neighbors. Here, we extended the two KNN methods of LSimpute, imputation by the nearest variables (KNN-V) and imputation by the nearest subjects (KNN-S), so that they could be used to impute the phenomic data with mixed types of variables. Furthermore, we developed a hybrid of these two methods using global variable/subject weights (KNN-H) and adaptive variable/subject weights (KNN-A).

#### Impute by nearest variables (KNN-V)

To extend the KNN imputation method to data with mixed types of variables, we used established statistical correlation measures between different data types to measure the distance among different types of variables. As described in Table 1, the phenomic data usually contain four types of variables – continuous (Con), binary (Bin), multi-class categorical (Cat) and ordinal (Ord). Table 2 lists correlation measures across different data types to construct the correlation matrix for KNN-V (Additional file 1 contains more detailed description):

**Table 2:** **Correlation measures between different types of variables**

**Variables**	**Con**	**Ord**	**Bin**	**Cat**
Con	Spearman	--	--	--
Ord	Polyserial	Polycoric	--	--
Bin	Point Biserial	Rank Biserial	Phi	--
Cat	Point Biserial extension	Rank Biserial extension	Cramer’s V	Cramer’s V

Spearman’s rank correlation (Con vs. Con): we use Spearman’s rank correlation to measure the correlation between two continuous variables. It is equivalent to compute Pearson correlation based on ranks: $\mathrm{r}=1-6\times \frac{{\displaystyle {\sum}_{\mathrm{i}=1}^{\mathrm{N}}}{\mathrm{d}}_{\mathrm{i}}^2}{\mathrm{N}\times \left({\mathrm{N}}^2-1\right)}$, where d_i_ is the rank difference of each corresponding observation and N is the number of subjects.

Point biserial correlation (Con vs. Bin) and its extension (Con vs. Cat): Point biserial correlation between a continuous variable X and a dichotomous variable Y (Y = 0 or 1) is defined as $\mathrm{r}=\frac{{\overline{\mathrm{X}}}_1-{\overline{\mathrm{X}}}_0}{{\mathrm{S}}_{\mathrm{X}}/\sqrt{{\mathrm{p}}_{\mathrm{Y}}\times \left(1-{\mathrm{p}}_{\mathrm{Y}}\right)}}$, where ${\overline{\mathrm{X}}}_1$ and ${\overline{\mathrm{X}}}_0$ represent the means of X given Y = 1 and 0 respectively, S_X_, the standard deviation of X and p_Y_, the proportion of subjects with Y = 1. Note that the point biserial correlation is mathematically equivalent to the Pearson correlation and there is no underlying assumption for Y. When Y is a multi-level categorical variable with more than two possible values, the point biserial correlation can be generalized, assuming Y follows a multinomial distribution and the conditional distribution of X given Y is normal [29]. It is implemented by the “biserial.cor” function in the “ltm” R package.

Rank biserial correlation (Ord vs Bin) and its extension (Ord vs Cat): The rank biserial correlation replaces the continuous variable X in point biserial correlation with ranks. To calculate the correlation between an ordinal and a nominal variable (binary or multi-class), we transform the ordinal variable into ranks and then apply rank biserial correlation or its extension for the calculation [30].

Polyserial correlation (Con vs Ord): Polyserial correlation measures the correlation between a continuous X and an ordinal variable Y. Y is assumed to be defined from a latent continuous variable η, generated with equal space and is strictly monotonic. The joint distribution of the observed continuous variable X and η is assumed to be bivariate normal. The Polyserial correlation is the estimated correlation between X and η and is estimated by maximum likelihood [31]. It is implemented by the “polyserial” function in the “polycor” R package.

Polychoric correlation (Ord vs Ord): Polychoric correlation measures correlation between two ordinal variables. Similar to the polyserial correlation described above, polychoric correlation estimates the correlation of two underlying latent continuous variables, which are assumed to follow a bivariate normal distribution [32]. It is implemented by the “polychor” function in the “polycor” R package.

Phi (Bin vs Bin): Phi coefficient measures the correlation between two dichotomous variables. The phi coefficient is the linear correlation of an underlying bivariate discrete distribution [33-35]. The Phi correlation is calculated as $\mathrm{r}=\sqrt{{\mathrm{X}}^2/\mathrm{N}}$, where N is the number of subjects and X^2^ is the chi-square statistic for the 2 × 2 contingency table of the two binary variables.

Cramer’s V (Bin vs Cat and Cat vs Cat): Cramer’s V measures correlation between two nominal variables with two or more levels. It is based on the Pearson’s chi-square statistic [36]. The formula is given by: $\mathrm{r}=\sqrt{\frac{{\mathrm{X}}^2}{\mathrm{N}\times \left(\mathrm{H}-1\right)}}$, where N is the number of subjects, X^2^ is the chi-square statistic for the contingency table and H is the number of rows or columns, whichever is less.

We note that all correlation measures in Table 2 are based on the classical Pearson correlation (some with additional Gaussian assumptions on the data) and as a result, the correlations from different data types are comparable in selecting K nearest neighbors. A corresponding distance measure could be computed as d = |1 − r|, where r is the correlation measures between pairwise variables. Given a missing value in the data matrix for variable x (missing on subject i), only the K nearest neighbors of x (denoted as y_1_ … y_K_) are included in the prediction model. In addition, none of y_1_, …, y_K_ is allowed to have missing values for the same subject as the missing value to be predicted. For each neighbour, a generalized linear regression model with single predictor is constructed: g(μ) = α + βy_k_ using available cases, where μ = E(x) and g(·) is the link function. The regression methods used for the imputation of different types of variables are listed in Table 3. Missing values could be imputed by ${\widehat{\mathrm{x}}}_{\mathrm{i}\left(\mathrm{k}\right)}={\mathrm{g}}^{-1}\left(\upalpha +{\upbeta \mathrm{y}}_{\mathrm{i}\mathrm{k}}\right)$. Finally, the weighted average of estimated impute values from the K nearest neighbors is used to impute the missing value of continuous data type. For nominal variables (binary or multi-class categorical), weighted majority vote from the K nearest neighbors is used. For ordinal variables, we treat the levels as positive integers (i.e. 1, 2, 3,…, q) and the imputed value is given by the rounded value of the weighted average.

**Table 3:** **Methods for aggregating imputation information of different data types from K nearest neighbors**

**Variables**	**Regression methods**	**Final imputed value**
Con	Linear regression	${\displaystyle \sum }{\mathrm{w}}_{\mathrm{k}}\widehat{{\mathrm{y}}_{\mathrm{k}}}/{\displaystyle \sum }{\mathrm{w}}_{\mathrm{k}}$
Ord	Ordinal logistic regression	$\min \left( \max \left(1,\left[{\displaystyle \sum }{\mathrm{w}}_{\mathrm{k}}\widehat{{\mathrm{y}}_{\mathrm{k}}}/{\displaystyle \sum }{\mathrm{w}}_{\mathrm{k}}\right]\right),\mathrm{q}\right)$
Bin	Logistic regression	Weighted majority vote
Cat	Multinomial logistic regression	Weighted majority vote

(q: number of level for ordinal variable).

#### Impute by nearest subjects (KNN-S)

The procedure of the KNN-S is generally the same as that of the KNN-V. Here, we borrow information from the nearest subjects, instead of variables. Thus, we will have mixed type of values within each vector (subject). We defined similarity of a pair of subjects by the Gower’s distance [37]. For each pair of subjects, it is the average of distance between each variable for the pair of subjects considered: ${\mathrm{d}}_{\mathrm{ij}}=\frac{{\displaystyle {\sum}_{\mathrm{v}=1}^{\mathrm{V}}}{\updelta}_{\mathrm{ij}\mathrm{v}}{\mathrm{d}}_{\mathrm{ij}\mathrm{v}}}{{\displaystyle {\sum}_{\mathrm{v}=1}^{\mathrm{V}}}{\updelta}_{\mathrm{ij}\mathrm{v}}}$, where d_ijv_ is the dissimilarity score between subject i and j for the v^th^ variable and δ_ijv_ indicates whether the v^th^ variable is available for both subject i and j; it takes the value of 0 or 1. Depending on different types of variable, d_ijv_ is defined differently: (1) for dichotomous and multi-level categorical variables, d_ijv_ = 0 if the two subjects agree on the v^th^ variable, otherwise d_ijv_ = 1; (2) the contribution of other variables (continuous and ordinal) is the absolute difference of both values divided by the total range of that variable [37]. The calculation of the Gower’s distance is implemented by the “daisy” function in the “cluster” R package.

#### Hybrid imputation by nearest subjects and variables (KNN-H)

Since the nearest variables and the nearest subjects often both contain information to improve imputation, we propose to combine imputed values from KNN-S and KNN-V by:

$$ \mathrm{K}\mathrm{N}\mathrm{N}-\mathrm{H}=p\times \mathrm{K}\mathrm{N}\mathrm{N}-\mathrm{S}+\left(1-p\right)\times \mathrm{K}\mathrm{N}\mathrm{N}-\mathrm{V}. $$

Following Bø et. al. [28], we estimated p by simulating 5% secondary missing values in the dataset. Define a dataset (D_ij_)_NP_ with missing value indicator I_ij_ = 1 if missing and 0 other wise. We simulate second layer of missing values randomly (I_ij_’ = 1 if subject i variable j is missing at second layer), perform imputation and assess the normalized squared error of each imputed values using KNN-S and KNN-V(${\mathrm{e}}_{\mathrm{S}}^2$ and ${\mathrm{e}}_{\mathrm{V}}^2$). *p* is chosen to minimize

$$ {\displaystyle \sum }{\mathrm{e}}_{\mathrm{H}}^2={\displaystyle \sum }{\mathrm{p}}^2{\mathrm{e}}_{\mathrm{S}}^2+2\mathrm{p}\left(1-\mathrm{p}\right){\mathrm{e}}_{\mathrm{S}}\cdot {\mathrm{e}}_{\mathrm{V}}+{\left(1-\mathrm{p}\right)}^2{\mathrm{e}}_{\mathrm{V}}^2. $$

Thus, $\widehat{\mathrm{p}}= \min \left( \max \left(\frac{{\displaystyle \sum {e}_s^2-{\displaystyle \sum {e}_v{e}_s}}}{{\displaystyle \sum {e}_s^2-2{\displaystyle \sum {e}_v{e}_s+{\displaystyle \sum {e}_v^2}}}},0\right),1\right)$. We simulated second layer of missing values 20 times and estimated ${\widehat{p}}_i$ and took the average $\frac{{\displaystyle {\sum}_1^{20}}{\widehat{p}}_i}{20}$ as the estimate of p. Similar to KNN-V imputation, KNN-H imputed values are rounded to the closest integer for the ordinal variables and the weighted majority vote for nominal variables.

#### Hybrid imputation using adaptive weight (KNN-A)

Bø et. al. [28] observed that the log-ratios of the squared errors $\log \left({\mathrm{e}}_{\mathrm{v}}^2/{\mathrm{e}}_{\mathrm{s}}^2\right)$ was a decreasing function of r_max_ in microarray missing value imputation, where r_max_ is the correlation between the variable with missing value and its closest neighbour. Such a trend suggested that when r_max_ is larger, more weight should be given to KNN-V. Thus, p should vary for different r_max_. We adopted the same procedure to estimate the adaptive weight of p: we estimated p based on e_S_ and e_V_ within each sliding window of r_max_, (r_max_ − 0.1, r_max_ + 0.1), and require that at least 10 observations need to be extracted for the computation of p.

### Evaluation method

We compared different missing value imputation methods in both simulated data and real datasets. We evaluated the imputation performance by calculating root mean squared error (RMSE) for continuous and ordinal variables and proportion of false classification (PFC) for nominal variables. The pure simulated data are discussed in Simulated datasets below. For real datasets, we first generated the complete dataset (CD) from the original raw dataset (RD) with missing values. We then simulated missing values (e.g. randomly at 5% missing rate) to obtain the dataset with missing values (MD), performed imputation on the MD and assessed the performance by calculating the RMSE between the imputed and the real values. The squared errors are defined as ${e}^2=\frac{{\left({\widehat{y}}_{ij}-{y}_{ij}\right)}^2}{var\left({y}_j\right)}$ for continuous variables (*ŷ*_*ij*_ and *y*_*ij*_ are the imputed and the true values for subject i and variable j and var(*y*_*j*_) is the variance for variable *j*), ${e}^2={\left(\frac{{\widehat{y}}_{ij}-{y}_{ij}}{p-1}\right)}^2$ for ordinal variables (p is the number of possible levels of *y*_*j*_), and *e*^2^ = χ(*ŷ*_*ij*_ ≠ *y*_*ij*_) for nominal variables (χ(⋅) is an indicator function). The RMSE for continuous and ordinal variables is defined as $\sqrt{ave\left({e}^2\right)}$ and the PFC for nominal variables is *ave*(*e*). We estimated the RMSE and the PFC by 20 randomly generated MDs.

### Simulated datasets

Simulation of complete datasets (CD): To demonstrate the performance of various methods under different correlation structure, we considered three scenarios to simulate N = 600 subjects and P = 300 variables.

**Figure 1:**
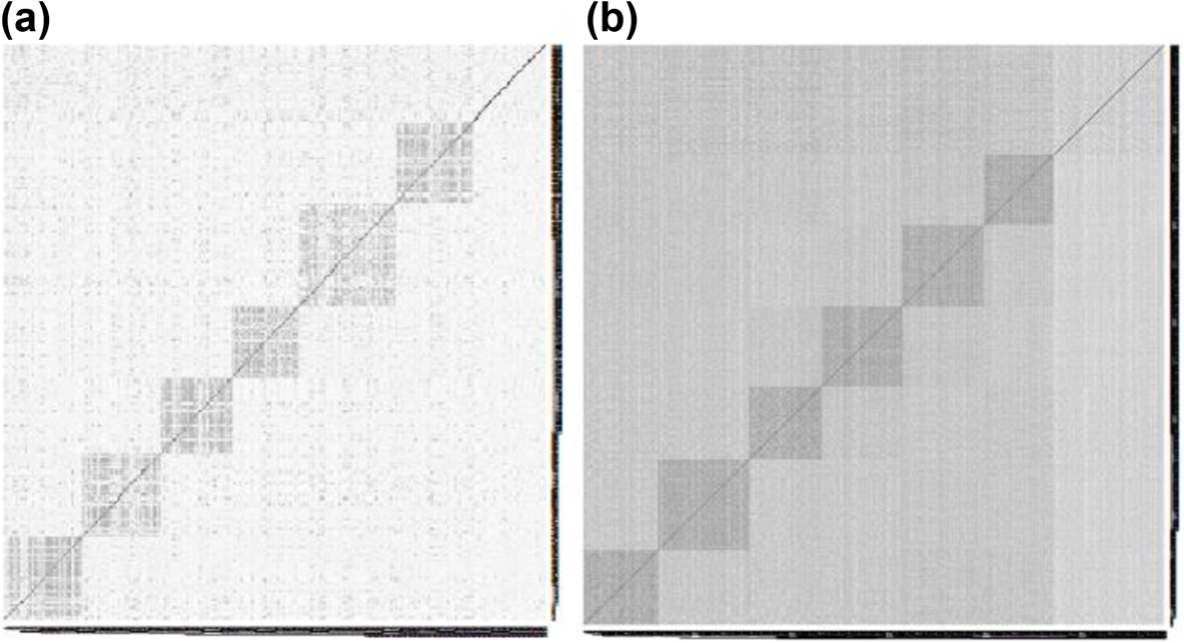
**Heatmap of distance matrix in simulation I. (a)** Variable and **(b)** Subject distance matrixes of Simulation I. (black: small distance/high correlation; white: large distance/low correlation).

Simulation I (six variable clusters + six subject clusters): We first generated the number of subjects in each cluster from Pois(80), and number of variables in each cluster from Pois(40). To create the correlation structure among variables, we first generated a common basis δ_i_ (i =1…6) with length N for variables in cluster i from N(μ, 4), where μ is randomly sampled from UNIF(−2, 2). Then we generated a set of slope and intercept (α_ip_, β_ip_), p = 1… v_i_, so that each variable is a linear transformation of the common basis and therefore the correlation structure is preserved. The rest of the variables which were independent of those grouped variables were random samples from N(0, 4). The subject correlation structure was generated following the similar strategy: we first generated common basis γ_j_ (j =1…6) from N(1,2) with length P. For all subjects in cluster j, γ_j_ was added to each of them to create correlation within subjects. And the rest of subjects were generated from N(0, 4 × I_P × P_). To create data of mixed types, we randomly converted 100 variables into nominal variables and 60 variables into ordinal variables by randomly generating 3 to 6 ordinal/nominal levels. The proportions of different variable types were similar to that of the COPD data set. The heatmaps of subject and variable distance matrixes of the simulated data are shown in Figure 1.

Simulation II (twenty variable groups + twenty subject groups): The number of clusters is increased to 20. The numbers of subjects in each cluster were generated from Pois(25) and the numbers of variables in each cluster were from Pois(15) (Additional file 1: Figure S1).

Simulation III (No variable groups + forty subject groups): In this simulation, we generated data with sparse between-variable correlation but strong between-subject correlations, a setting similar to the nominal variables in the SARP data set (Additional file 1: Figure S6(c)). The number of subjects in each cluster followed Pois (14). In each subject cluster, a common base γ_c_ (c =1…40) with length P were shared, and was added by a random error from N(0, 0.01). We created sparse categorical variable by cutting continuous variable at the extreme quantiles (≤ 5 % or ≥ 95 %) and generated the other cutting point randomly from UNIF(0.01, 0.99) which created up to 30 levels. (Additional file 1: Figure S2).

Generate datasets with missing values (MD) from complete data (CD): MD were generated by randomly removing m% values from simulated CD described above or CD from real data described in Section Real data. We considered m% = 5%, 20%, 40% in our simulation studies. All three settings were repeated for 20 times.

### Imputability measure

Current practice in the field is to impute all missing data after filtering out variables or subjects with more than a fixed percent (e.g. 20%) of missing values. This practice implicitly assumes that all missing values are imputable by borrowing information from other variables or subjects. This assumption is usually true in microarray or other high-throughput marker data since genes usually interact with each other and are co-regulated at the systemic level. For high-dimensional phenomic data, however, we have observed that many variables do not associate or interact with other variables and are difficult to impute. Therefore, to identify these missing values, we introduce a novel concept of “imputability” and develop a quantitative “imputability measure” (IM). Specifically, given a dataset with missing values, we generate “second layer” of missing values as described above. We then perform the KNN-V and the KNN-S method on a “secondary simulated layer” of missing values. The procedure is repeated for t times (t =10 is usually sufficient) and E_i_ and E_j_ could be calculated as the average of the RMSEs for the second layer missing values of subject i (i = 1,…,N) and variable j (j = 1,…,P) of the t times of imputations. Let IMs_i_ = exp(−E_i_) and IMv_j_ = exp(−E_j_). The IM for a missing value Dij is defined as max(IMs_i_, IMv_j_). IM provides quantitative evidence of how well each missing value can be imputed by borrowing information from other variables or subjects. IM ranges between 0 and 1 and small IM values represent large imputation errors that should raise concerns of using imputation. Detailed Procedure of generating IM is described in Additional file 2 algorithm 1. In the application guideline to be proposed in the Result section, we will recommend users to avoid imputation or impute with caution for missing values with IM less than a pre-specified threshold.

### The self-training selection (STS) scheme

**Figure 2:**
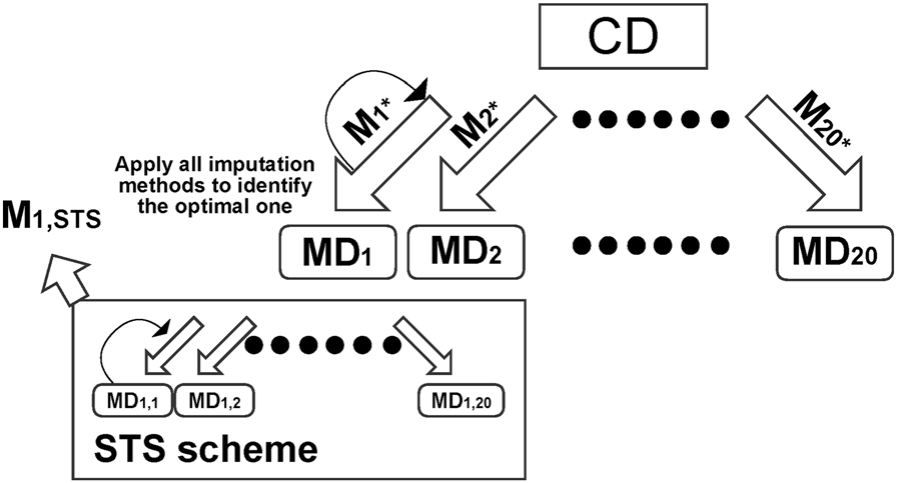
**Diagram of evaluating performance of STS scheme in a real complete data set (CD).** Missing data sets are randomly generated for 20 times (MD_1_, ⋅⋅⋅, MD_20_). The STS scheme is applied to learn the best method from STS simulation (denoted as M_b,STS_ for the b-th missing data set MD_b_). The true best (in terms of RMSE) method for MD_b_ is denoted as M_b*_ and the STS best (in terms of RMSE across MD_b,1_, …, MD_b,20_) method is denoted as M_b,STS_. When M_b,STS_ = M_b*_, the STS scheme successfully selects the optimal method.

In our analyses, no imputation method performed universally better than all other methods. Thus, the best choice of imputation method depends on the particular structure of a given data. Previously, we proposed a Self-Training Selection (STS) scheme for microarray missing value imputation [24]. Here we applied the STS scheme and evaluated its performance in the complete real datasets. Figure 2. shows a diagram of the STS scheme and how we evaluated the STS scheme. From a CD, we simulated 20 MDs (MD_1_, MD_2_, …, MD_20_). Our goal was to identify the best method for the data set. To achieve that, we randomly generated a second layer of missing values within each MD_b_ (1 ≤ b ≤ 20) for 20 times and denoted the data sets with two layers of missing values as MD_b,i_ (1 ≤ i ≤ 20). The method that performs the best in the second layer missing values imputation, i.e., generate the smallest average RMSE, was identified as the method selected by the STS scheme for missing value imputation of MD_b_ (denoted as M_b, STS_). Consider the optimal method identified by the first layer STS as the “true” optimal imputation method, denoted as M_b*_, we counted how many times of the 20 simulations that M_b, STS_ = M_b*_ (i.e. ${\displaystyle {\sum}_{\mathrm{b}=1}^{20}\mathrm{I}\left({\mathrm{M}}_{\mathrm{b},\mathrm{S}\mathrm{T}\mathrm{S}}={\mathrm{M}}_{\mathrm{b}*}\right)}$/20, where I(⋅) is the indicator function) as the accuracy of STS scheme.

## Results

### Simulation results

We compared the performance of seven methods – mean imputation (MeanImp), KNN-V, KNN-S, KNN-H, KNN-A, missForest and MICE – on the three simulation scenarios described above. When implementing MICE, the R packages returned errors when the nominal or ordinal variables contained large number of levels and any level contained a small number of observations. As a result, MICE was not applied to Simulation III evaluation. We first performed simulation to determine effects on the imputation by the choice of K. We tested K = 5, 10 and 15 for missing value = 5%, 10% and 20% on different types of data. The imputation results with different K values are similar (see Additional file 1: Figure S3). We thus chose K = 5 for both simulation and real data applications as it generated good performance in most situations.

**Figure 3:**
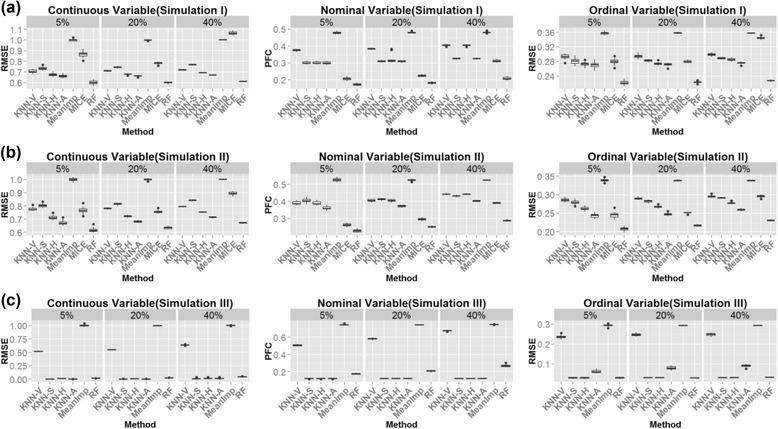
**Boxplots of RMSE/PFC for (a) Simulation I and (b) Simulation II and (c) Simulation III.** KNN-based methods: KNN-V, KNN-S, KNN-H and KNN-A; RF: MissForest algorithim; MICE: multivariate imputation by chained equations; MeanImp: mean imputation.

Figure 3 shows the boxplots of the RMSEs of the three types of variables from 20 simulations for the three simulation scenarios. For simulation I and II, we observed that missForest performed the best in all three data types. MICE performed better than the KNN-methods in nominal missing imputation, but performed worse in the imputation of continuous and ordinal variables. The two hybrid KNN methods (KNN-A and KNN-H) consistently performed better than KNN-V and KNN-S, showing the effectiveness to combine information from variables and subjects. KNN-A performed slightly better than KNN-H especially in the first two simulation scenarios, indicating the advantages of adaptive weight in combining KNN-V and KNN-S information. For simulation III, KNN-S performed overall the best while KNN-V failed. This is expected due to the lack of correlation between variables. missForest was also not as good as KNN-S in the continuous and nominal variable imputations. In this case, the performance of KNN-S, KNN-H and KNN-A were not affected much by missing percentages, due to the strong correlation among subjects.

### Real data applications

**Figure 4:**
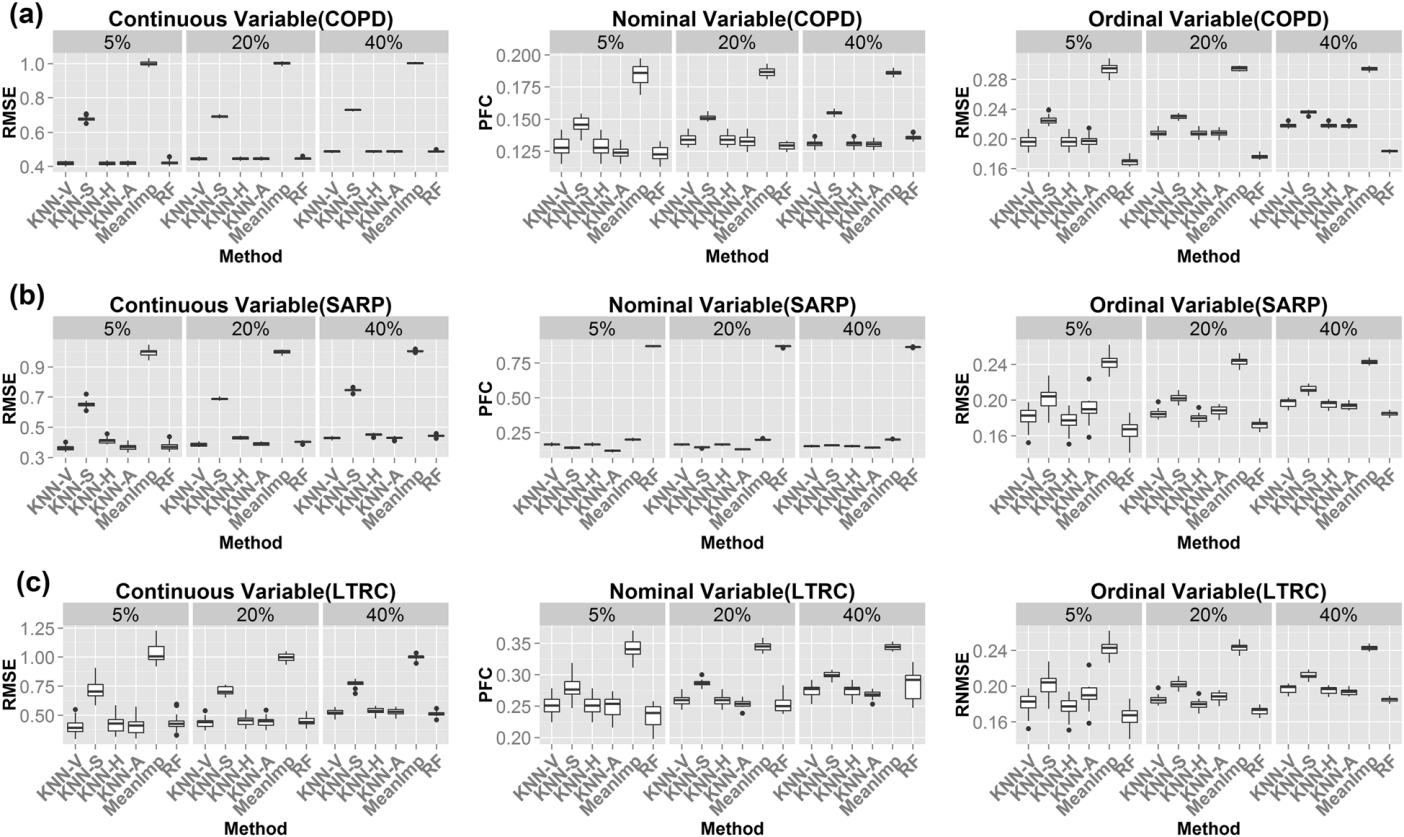
**Boxplots of RMSE/PFC for (a) COPD; (b) SARP and (c) LTRC.** KNN-based methods: KNN-V, KNN-S, KNN-H and KNN-A; RF: MissForest algorithm; MeanImp: Mean imputation.

Next we compared different methods in three real datasets. Similar to the above simulation study, we first investigate the choice of K for the simulation of real data sets and reached the same conclusion (Additional file 1: Figure S4). In order to implement MICE in our comparative analysis, we had to remove categorical variables with any sparse level (i.e. having <10% of the total observations) and those with greater than 10 levels. The numbers of variables after such filtering are shown in Additional file 1: Table S1. Since only 26% (38/144), 14% (16/118) and 45% (49/108) of nominal and ordinal variables were retained after the filtering, we decided to remove MICE from the comparison and report the comparative results of the remaining methods with the unfiltered data in Figure 4. The comparative results for all methods including MICE on the filtered data are available in Additional file 1: Figure S5. As expected, the mean imputation almost always performed the worst (Figure 4). KNN-V usually performed better than KNN-S (except for the nominal variables in SARP), indicating better information borrowed from neighboring variables than subjects. The hybrid methods KNN-H and KNN-A performed better than either KNN-S or KNN-V alone. KNN-A seemed to slightly out performed KNN-H. missForest was usually the best performer with an exception of nominal variables in the SARP data set. This is probably because of the low mutual correlation of nominal variables with other variables in this data set as demonstrated in Additional file 1: Figure S6. (note that missForest only borrows information from variables). Overall, no method universally outperformed other methods. In Additional file 1: Figure S5 after filtering, the comparative result is similar to Figure 4 for KNN methods and missForest. The MICE method had unstable performance: sometimes performs among the best and sometimes much worse than all the others.

### Imputability measure

**Figure 5:**
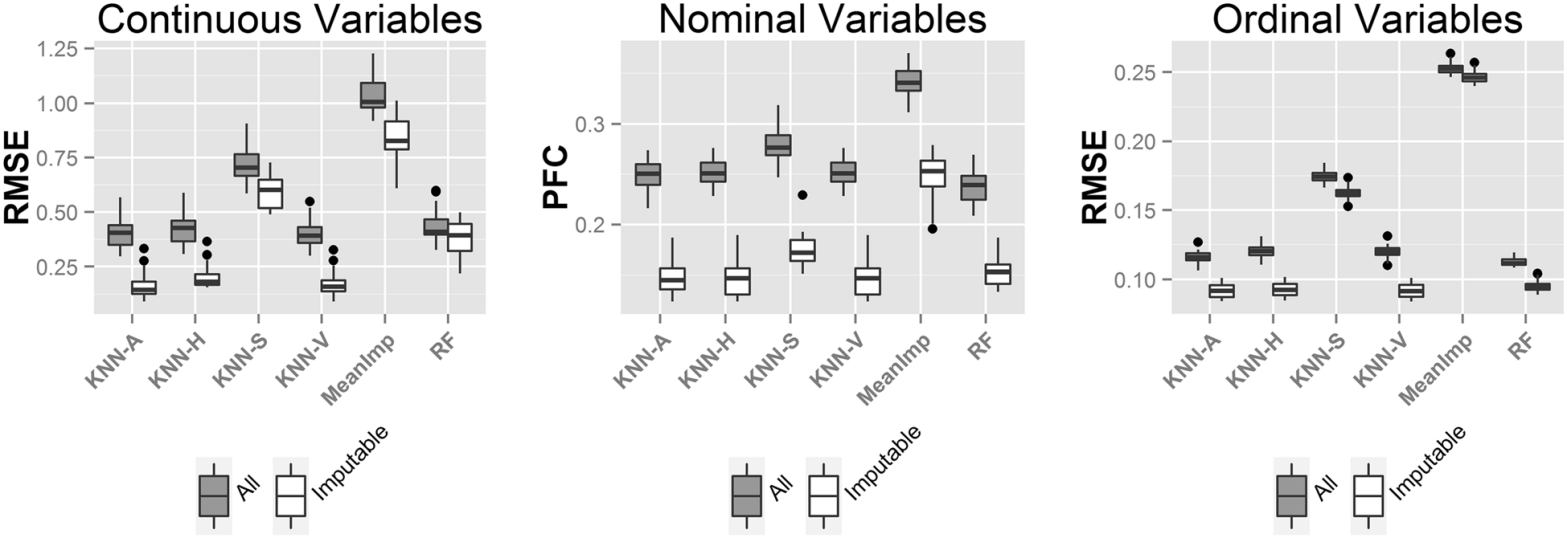
**Boxplots of RMSE/PFC evaluated using (1) all imputed values and (2) only imputable values in LTRC dataset.** Boxplots of RMSE/PFC evaluated using (1) all imputed values and (2) only imputable values in LTRC dataset with m =5% missingness. Color: grey (evaluation using all imputed values); white (evaluation using only imputable values).

The motivation of imputability concept rests in that some variables or subjects have no near neighbour to borrow information from, hence cannot be imputed accurately. The distribution of imputability measure (IM; defined in Section Imputability measure) of the variables (IMv) and subjects (IMs) of COPD, LTRC and SARP data are shown in Additional file 1: Figure S7. We observed a heavy tail to the left, which indicated existence of many un-imputable subjects and variables. By including these poorly imputed values, we risk to reduce the accuracy and power of downstream analyses. To demonstrate the usefulness of IM, we compared the RMSE/PFC before and after removing un-imputable values. Figure 5 shows significant reduction of RMSE and PFC by removing missing values with the lowest 25% IMs. In Additional file 1: Figure S8, heatmaps of IMs for the three real datasets are presented. Values colored in green are with low IMs and should be imputed with caution.

### The self-training selection scheme (STS) and an application guideline

**Figure 6:**
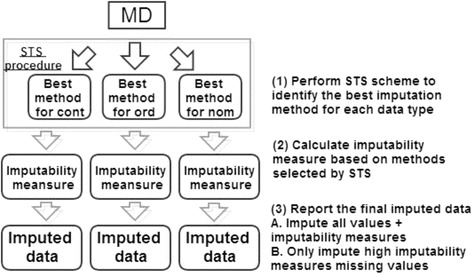
**An application guideline to apply the STS scheme for a real dataset with missing values.**

Finally, we applied the STS scheme to the real datasets and the performance is reported in Table 4. Methods with RMSE difference within 5% range are considered comparable. Thus, if a method generates RMSE within 5% of the minimum RMSE of all methods, we considered the method not distinguishable from the optimal method and the method is also an optimal choice. We found that the STS scheme can almost always select the true optimal missing value imputation method with perfect accuracy (with only several exceptions down to 75%-95% accuracy). Figure 6 describes an application guideline for the phenomic missing value imputation. Firstly, the STS scheme is applied to the MD of different data types separately to identify the best imputation method. The IMs are then calculated based on the selected optimal method. Finally, imputation is performed based on the optimal method selected by the STS scheme and the users have two options to move on to downstream analyses. For Option A, all missing values are imputed accompanied by IMs that can be incorporated in downstream analyses. In Option B, only missing values with IMs higher than a pre-specified threshold are imputed and reported.

**Table 4:** **Accuracy of STS in real data applications**

**Data**	**m%**	**Continuous variables**		**Nominal variables**		**Ordinal variables**	
		**Predicted optimal method (No. of time selected)**	**Accuracy**	**Predicted optimal method (No. of time selected)**	**Accuracy**	**Predicted optimal method (No. of time selected)**	**Accuracy**
COPD	5%	KNN-V(10), RF(10)	100%	RF(10), KNN-A(8), KNN-V(2)	100%	RF(20)	100%
	20%	KNN-V(13), RF(6), KNN-H(1)	100%	RF(14), KNN-A(4), KNN-V(2)	100%	RF(20)	100%
	40%	KNN-V(10), RF(10)	100%	KNN-V(16), RF(1), KNN-A(3)	95%	RF(20)	100%
LTRC	5%	KNN-V(15), KNN-A(3), RF(2)	95%	RF(14), KNN-A(3), KNN-V(3)	75%	RF(19), KNN-A(1)	100%
	20%	KNN-V(12), RF(8)	85%	RF(15), KNN-V(1), KNN-A(4)	100%	RF(16), KNN-A(4)	100%
	40%	RF(13), KNN-V(7)	90%	KNN-A(13), RF(6), KNN-V(1)	100%	RF(20)	100%
SARP	5%	KNN-V(13), KNN-A(6), RF(1)	100%	KNN-A(20)	100%	RF(18), KNN-H(2)	100%
	20%	KNN-V(16), KNN-A(4)	100%	KNN-A(20)	100%	RF(16), KNN-H(4)	100%
	40%	KNN-V(17), KNN-A(3)	100%	KNN-A(20)	100%	RF(20)	100%

Note: Here “predicted optimal method” means the predicted method with minimal RMSE for second layer of missing values; and “accuracy” means the chances we correctly predict optimal method. ($\mathrm{Accuracy} = \frac{{\displaystyle {\sum}_{b=1}^{20}}I\left({M}_{b,STS}={M}_{b*}\right)}{20}\times 100\%$).

## Discussion

In our comparative study of the imputation methods available for phenomic data, MICE encountered difficulty in nominal and ordinal data types when any level in the variable has few observations. This limited its application to some real data. It also had unstable performance, with some situations among the top performers while in some other situations it performed much worse than the KNN methods and missForest. For the KNN methods, the hybrid methods (KNN-H and KNN-A) that combined information from neighboring subjects and variables usually performed better than borrowing information from either subjects (KNN-S) or variables (KNN-V) alone. missForest usually was among the top performers while it could fail when correlations among variables are sparse. In the proposed KNN-based methods, when there are lots of nominal variables with sparse levels, ordinary logistic regression will also fail to work. When this happen, contingency table is used to impute the missing values. This partly explained why across different missing percentage, (5% to 40%) the accuracy remained mostly unchanged. It is also due to the lack of similar variables with nominal missing values. Overall, no method universally performed the best in all situations. Thus, we implemented a STS scheme [24] previously developed for microarray missing value imputation to identify the best method for phenomic data. Our evaluation showed that STS selected the true best method with almost perfect accuracy.

In missing value imputation of microarray data, it is a common practice to impute all missing values and return a complete data matrix for down-stream analyses. In our analysis, we, however, found that many variables or subjects are intrinsically difficult to impute in phenomic data. Our proposed IM was found effective in identification of missing values that intrinsically cannot be imputed well and improved the imputation performance. As a result, our application guideline recommended to always report both the imputed values and IMs when all missing values were imputed (option A) or only to impute missing values with high IMs (option B). In the former output, it is possible to incorporate the IM values in downstream analyses (e.g. by down-weighting imputed values in the analysis with low IMs).

We note that RMSE has been used to evaluate performance of different methods in this paper. Depending on the final biological objectives, there are many choices of downstream analyses after imputation; for example, association analysis, cluster analysis, classification analysis, pathway enrichment analysis and graphical models, to name a few. While the impact of imputation methods to these downstream analyses is the ultimate interest, it is beyond the scope of this paper. We decided that RMSE is the most direct assessment that we could use to evaluate the methods. In our simulation and real data, we examined data size of hundreds of clinical variables and hundreds of samples. This is a common scale of phenomic datasets we usually expect. In the future, if larger scale of variables or patients are expected (e.g. up to thousands), more evaluations on the methodological and computational capabilities of different methods will be needed.

With the accelerated pace of phenomic data generation in many complex diseases nowadays, missing values are almost always inevitable. Ignoring subjects or variables with any missing value is no longer practical as it significantly reduces the statistical power and may distort the conclusion. Missing value imputation is a practical and powerful solution while such a practice in high-dimensional phenomic data has not drawn much attention in the literature. To our knowledge, our pipeline is the first complete guideline to the missing value imputation in high-dimensional phenomic data. We believe that the methods, the imputability concept, the STS scheme and the application guideline we proposed in this paper will provide practical guidance to researchers in the field.

## Conclusions

In this paper, we conducted comprehensive comparison of existing imputation methods for phenomic data, including four variations of KNN imputation methods developed by us in this paper, missForest and MICE, using three simulation scenarios and three phenomic real datasets. We proposed a novel “imputability” concept with a quantitative imputability measure (IM) to characterize whether a missing value is imputable or not. More importantly, since the choice of the best imputation method depends on different data types and data structure, we implemented a simulation-based “self-training selection” (STS) scheme to select the best methods in a given application. Finally, we illustrated an application guideline for practitioners to apply to real phenomic applications. The R package “phenomeImpute” is available to implement all methods and the analytical pipeline proposed in this paper.

### Availability of supporting data

The R package “PhenomeImpute” is available in the webpage http://tsenglab.biostat.pitt.edu/software.htm. Three real datasets and R codes are available in http://tsenglab.biostat.pitt.edu/publication.htm.

## Additional files

Additional file 1: **Supplementary materials.** This file contains supplementary figures, tables and detailed description of correlation measures. **Figure S1.** Heatmaps of (a) Variable (b) Subject distance in Simulation II. **Figure S2.** Heatmaps of (a) Variable (b) Subject distance in Simulation III. **Figure S3.** Selection of *K* for KNN-S (A) and KNN-V (B). First row: Simulation I; Second row: Simulation II; Third row: Simulation III. **Figure S4.** Selection of *K* for KNN-S (A) and KNN-V (B). First row: COPD; Second row: LTRC; Third row: SARP. **Figure S5.** Comparison of different missing value imputation methods in filtered data such that MICE can be implemented (First row: COPD; Second row: LTRC; Third row: SARP). **Figure S6.** Heatmaps of variable distance matrix (above) and subject distance matrix (below) of real data (COPD/LTRC/SARP). **Figure S7.** Density of IMv and IMs for three real datasets. **Figure S8.** Heatmaps of imputability measures for (a)COPD;(b)LTRC;(c)SARP. Red indicates larger imputability measures; green indicates smaller imputability measures. Detailed description of correlation measures. Table S1. Number of variables after filtering out sparse ordinal or nominal variables for MICE implementation.

Additional file 2: **Algorithm 1.** Procedure of generating Imputability Measure (IM).
