# Supplementary material for: Missing value imputation in high-dimensional phenomic data: imputable or not, and how?
Source: BMC Bioinformatics. 2014 Nov 5;15(1):346. doi: 10.1186/s12859-014-0346-6 (PMC4228077; doi:10.1186/s12859-014-0346-6)
Supplement: Supplementary file 1 — Supplementary materials. This file contains supplementary figures, tables and detailed description of correlation measures. Figure S1. Heatmaps of (a) Variable (b) Subject distance in Simulation II. Figure S2. Heatmaps of (a) Variable (b) Subject distance in Simulation III. Figure S3. Selection of K for KNN-S (A) and KNN-V (B). First row: Simulation I; Second row: Simulation II; Third row: Simulation III. Figure S4. Selection of K for KNN-S (A) and KNN-V (B). First row: COPD; Second row: LTRC; Third row: SARP. Figure S5. Comparison of different missing value imputation methods in filtered data such that MICE can be implemented (First row: COPD; Second row: LTRC; Third row: SARP). Figure S6. Heatmaps of variable distance matrix (above) and subject distance matrix (below) of real data (COPD/LTRC/SARP). Figure S7. Density of IMv and IMs for three real datasets. Figure S8. Heatmaps of imputability measures for (a)COPD;(b)LTRC;(c)SARP. Red indicates larger imputability measures; green indicates smaller imputability measures. Detailed description of correlation measures. Table S1. Number of variables after filtering out sparse ordinal or nominal variables for MICE implementation. [file 12859_2014_346_MOESM1_ESM.docx]

Supplementary material: Additional file 1

Missing value imputation in high-dimensional phenomic data: Imputable or not? And how?

Serena G. Liao^1,*^, Yan Lin^1,*^, Dongwan D. Kang^1^, Naftali Kaminski^4^, Frank C. Sciurba^5^, George C. Tseng^1,2,3,§^

**
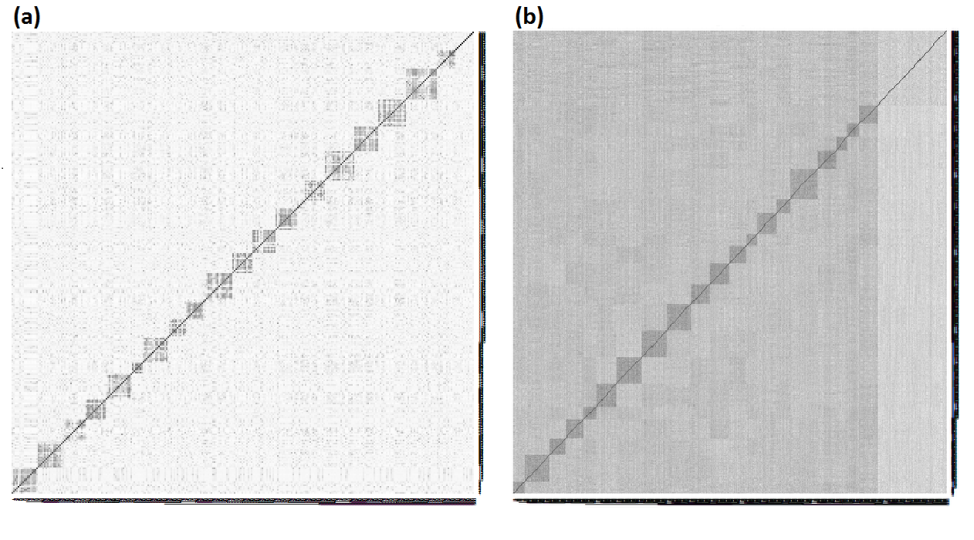
**

**Supplement Figure 1**. Heatmaps of (a) Variable (b) Subject distance in Simulation II: twenty variable clusters and twenty subject clusters. Black: small distance/high correlation; white: large distance/low correlation.


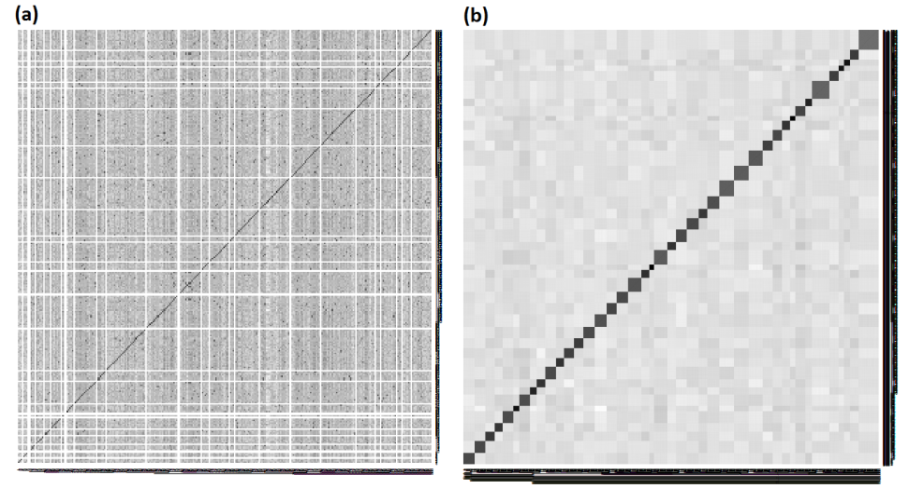


**Supplement Figure 2.** Heatmaps of (a) Variable (b) Subject distance in Simulation III: no variable clusters and forty subject clusters. Black: small distance/high correlation; white: large distance/low correlation.

(A)


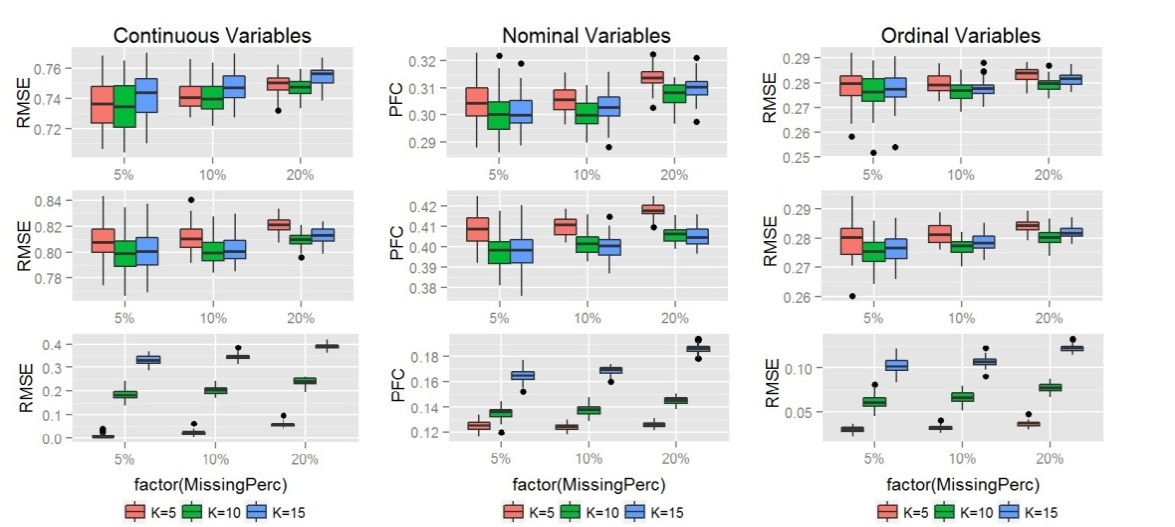


**(B)**


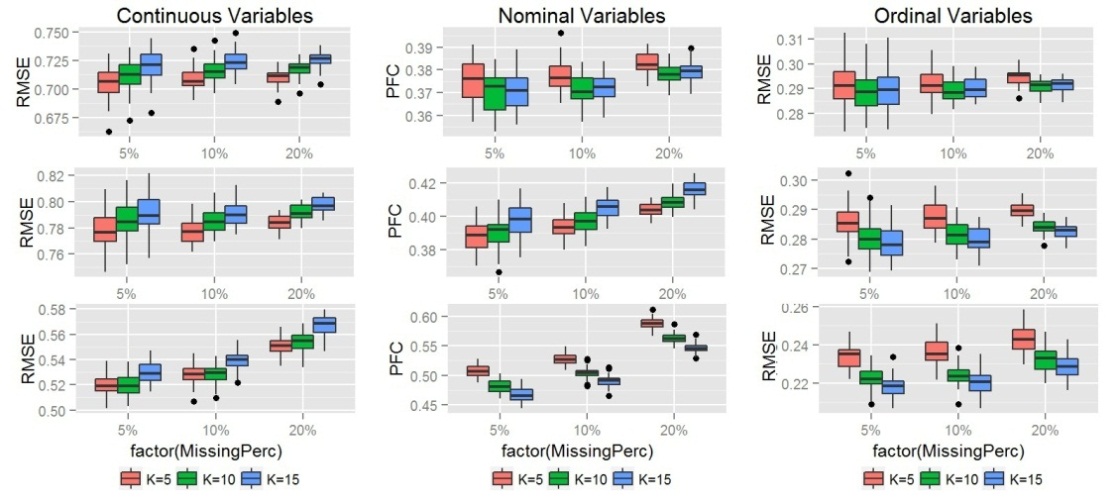


**Supplement Figure 3.** Selection of *K* for KNN-S (A) and KNN-V (B). First row: Simulation I; Second row: Simulation II; Third row: Simulation III. There is no overall best selection of *K* across all settings. We select *K*=5 for its good performance in most cases and convenience.

(A)
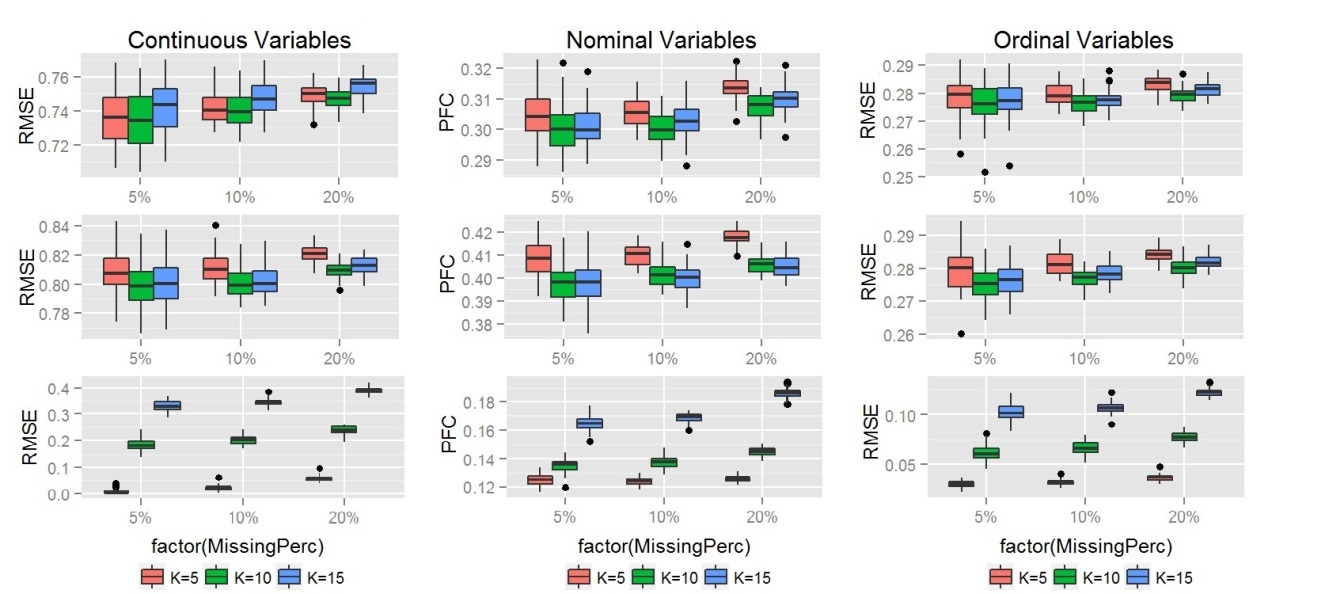


**(B)**

**
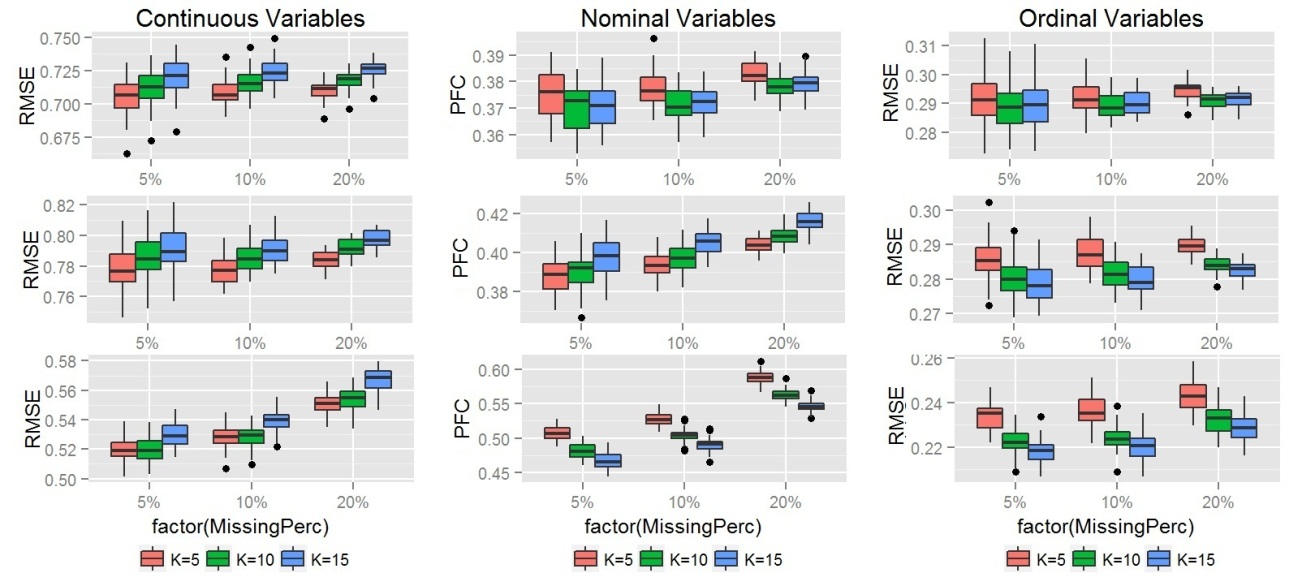
**

**Supplement Figure 4.** Selection of *K* for KNN-S (A) and KNN-V (B). First row: COPD; Second row: LTRC; Third row: SARP. There is no overall best selection of *K* across all settings. We select *K*=5 for its good performance in most cases and convenience.


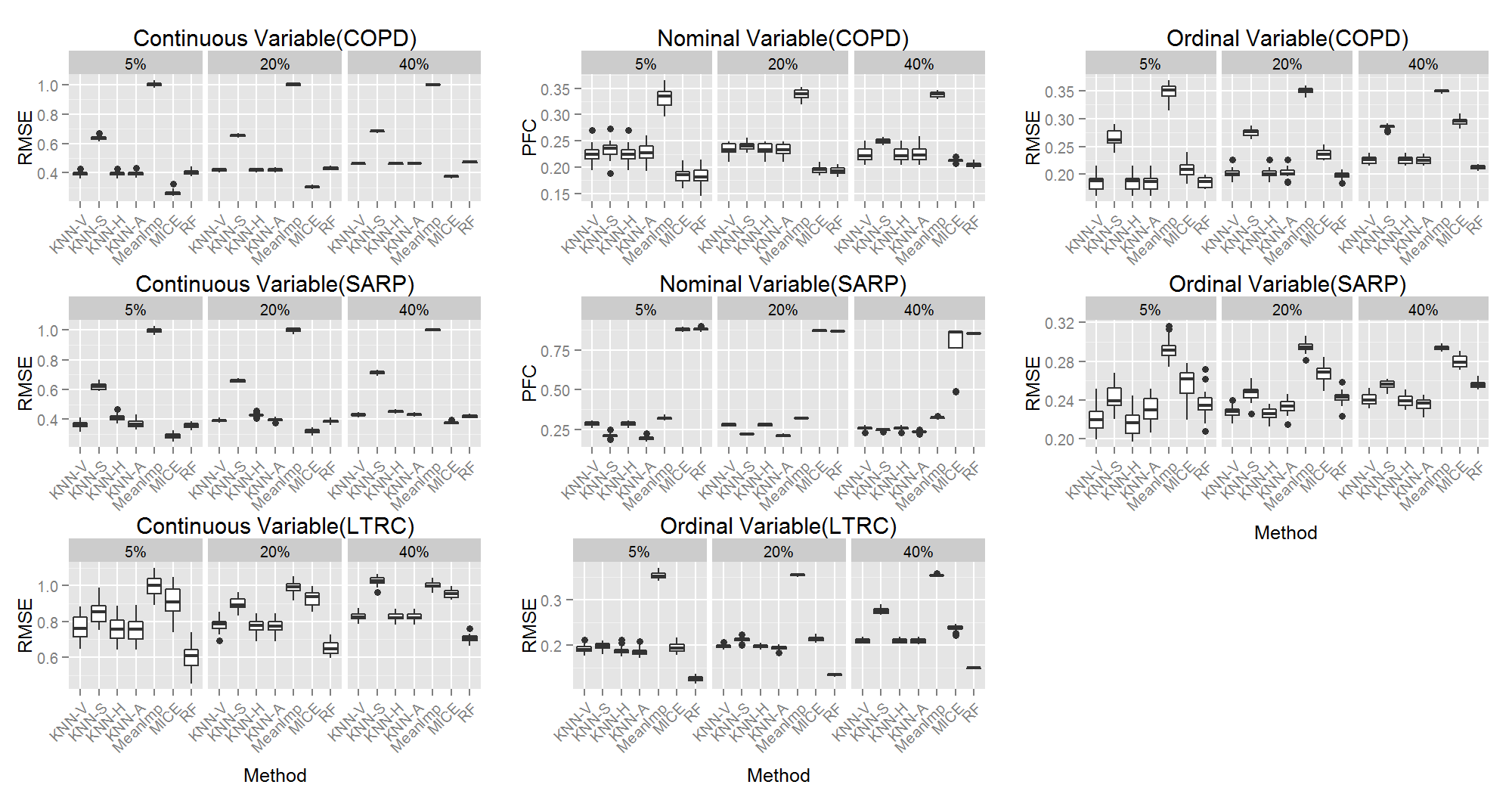


**Supplement Figure 5.** Comparison of different missing value imputation methods in filtered data such that MICE can be implemented (First row: COPD; Second row: LTRC; Third row: SARP). The number of variables in the filtered data is much smaller than the original data (see Table S1).


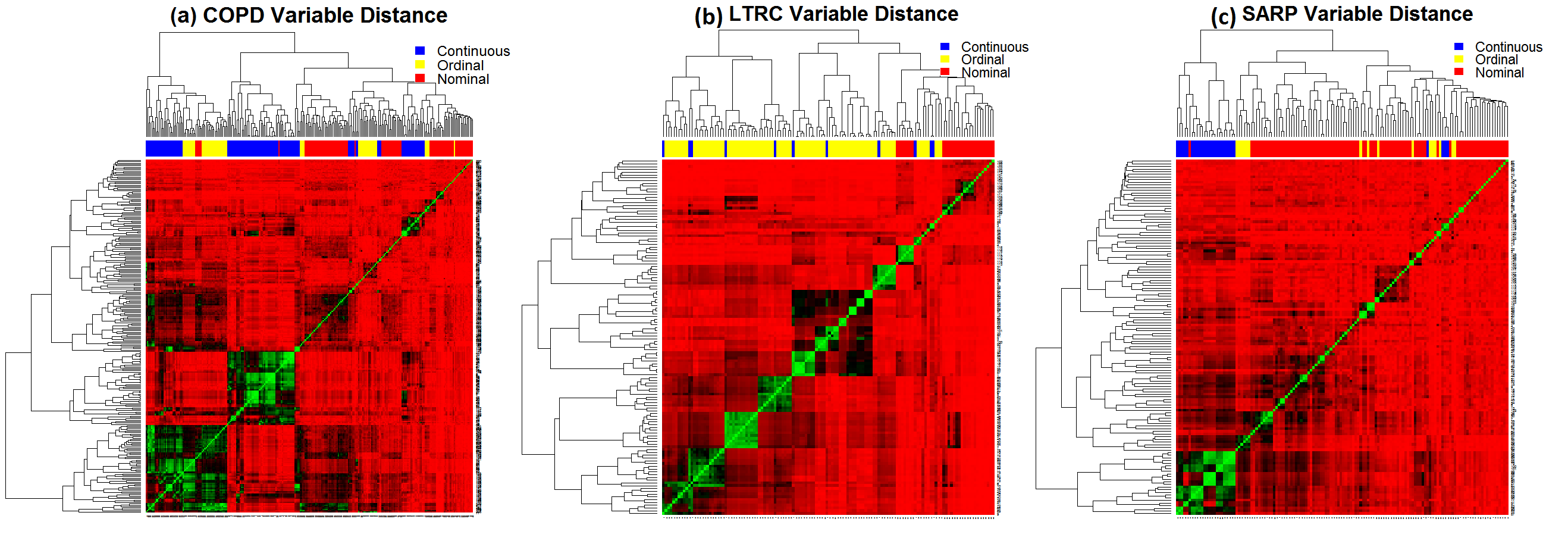


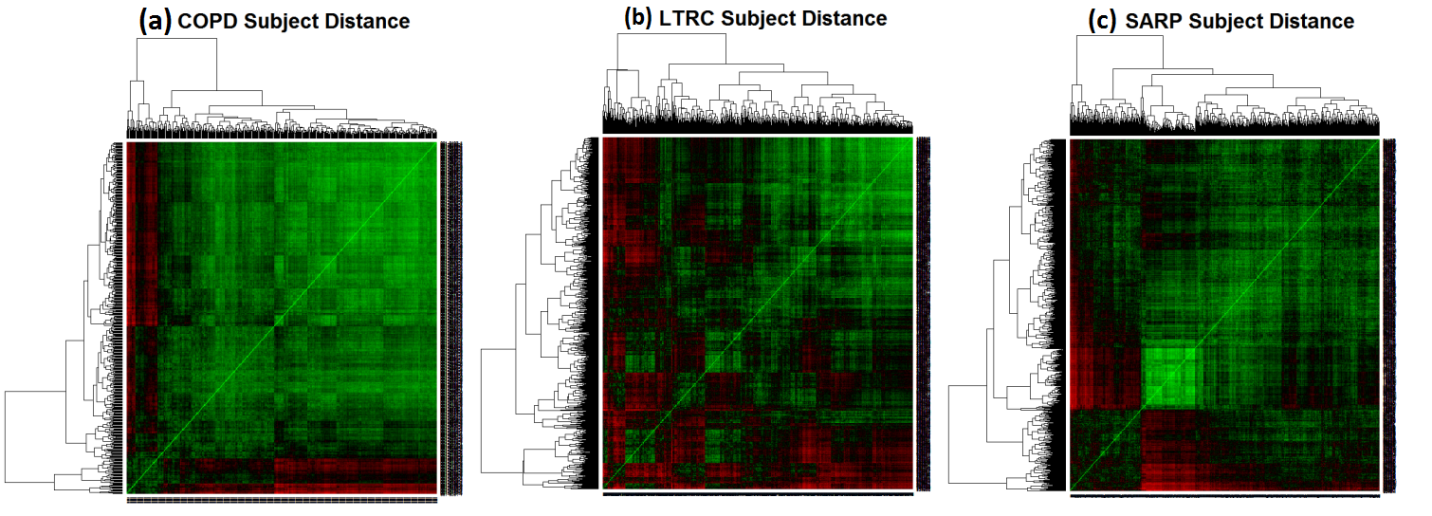


**Supplement Figure 6.** Heatmaps of variable distance matrix (above) and subject distance matrix (below) of real data (COPD/LTRC/SARP): (green: smaller distance; red: larger distance)


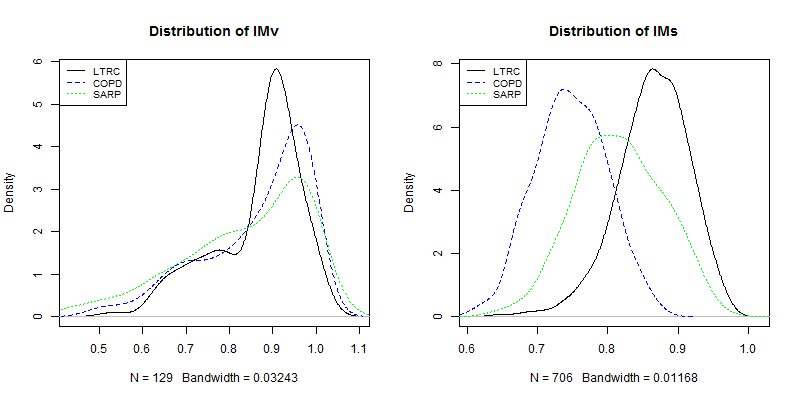


**Supplement Figure 7.** Density of IMv and IMs for three real datasets.


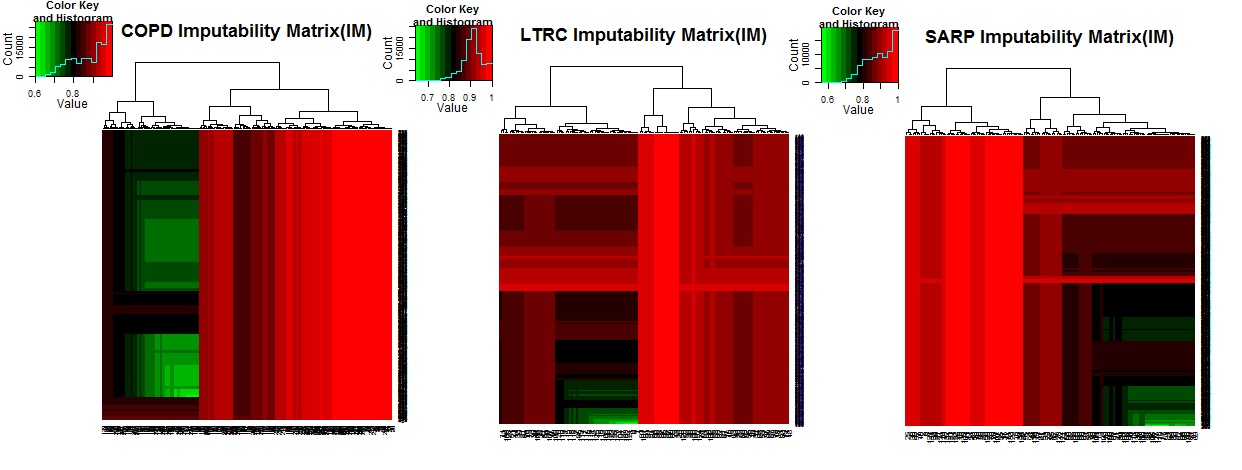


**Supplement Figure 8.** Heatmaps of imputability measures for (a)COPD;(b)LTRC;(c)SARP. Red indicates larger imputability measures; green indicates smaller imputability measures.

**Table S2.** Comparison of computation time for proposed methods with missForest when m=5%. (min/simulation)

| Data | KNN-V | KNN-S | KNN-H | KNN-A | missForest |
| --- | --- | --- | --- | --- | --- |
| COPD | 6.01 | 0.15 | 32.12 | 35.76 | 20.20 |
| LTRC | 4.82 | 0.17 | 20.20 | 24.43 | 7.43 |
| SARP | 3.82 | 0.12 | 18.77 | 20.54 | 7.25 |

**Table S1.** Number of variables after filtering out sparse ordinal or nominal variables for MICE implementation.

| Data | Continuous | Nominal  (binary and categorical) | Ordinal | Total |
| --- | --- | --- | --- | --- |
| COPD | 113 | 25 | 13 | 151 |
| LTRC | 11 | 0 | 16 | 27 |
| SARP | 27 | 41 | 8 | 76 |
