# Supplementary material for: Missing value imputation in high-dimensional phenomic data: imputable or not, and how?
Source: BMC Bioinformatics. 2014 Nov 5;15(1):346. doi: 10.1186/s12859-014-0346-6 (PMC4228077; doi:10.1186/s12859-014-0346-6)
Supplement: Supplementary file 2 — Algorithm 1. Procedure of generating Imputability Measure (IM). [file 12859_2014_346_MOESM2_ESM.docx]

Supplementary material: Additional file 2

Missing value imputation in high-dimensional phenomic data: Imputable or not? And how?

Serena G. Liao^1,*^, Yan Lin^1,*^, Dongwan D. Kang^1^, Naftali Kaminski^4^, Frank C. Sciurba^5^, George C. Tseng^1,2,3,§^

**Algorithm 1** Procedure of generating Imputability Measure (IM)

1: Given MD, generate a 5% secondary layer of missing value {$D_{i_{1},j_{1}}^{(t)},\ldots, D_{i_{Z},j_{Z}}^{(t)}$}, where t represents the t^th^ repetition. Z is the total number of missing values in the second layer.

2: Perform KNN-V and KNN-S to obtain the imputed values {$\hat{D}_{i_{1},j_{1}}^{(t,KNN-V)},\ldots, \hat{D}_{i_{Z},j_{Z}}^{(t,KNN-V)}$} and $\hat{D}_{i_{1},j_{1}}^{(t,KNN-S)},\ldots, \hat{D}_{i_{Z},j_{Z}}^{(t,KNN-S)}$}, go back to step 1 until T repetitions are finished

3: Calculate RMSE per variable for KNN-V {$E_{1}^{(v)},\ldots,E_{P}^{(v)}$} and RMSE per subject for KNN-S {$E_{1}^{(s)},\ldots,E_{N}^{(s)}$} as shown in Table 3.

4: Transform {$E_{1}^{(s)},\ldots,E_{N}^{(s)}$} and {$E_{1}^{(v)},\ldots,E_{P}^{(v)}$} to {${IM}_{1}^{(s)},\ldots,{IM}_{N}^{(s)}$} and {${IM}_{1}^{(v)},\ldots,{IM}_{P}^{(v)}$} as formula (5) and (6) in main text.

5: IM for a missing value at $D_{ij}$ is defined as $max({IM}_{i}^{\left( s \right)},{IM}_{j}^{\left( v \right)})$.
